# Mesenchymal Stromal Cell-Derived Extracellular Vesicles as a Therapeutic Treatment for Osteosarcopenia: Crosstalk Among Neurons, Muscle, and Bone

**DOI:** 10.3390/ijms26167875

**Published:** 2025-08-15

**Authors:** Martina Gatti, Francesca Beretti, Marta Malenchini, Emma Bertucci, Eleonora Ceneri, Matilde Y. Follo, Tullia Maraldi

**Affiliations:** 1 Department of Biomedical, Metabolic and Neural Sciences, University of Modena and Reggio Emilia, 41124 Modena, Italy; martina.gatti@unimore.it (M.G.); francesca.beretti@unimore.it (F.B.); marta.malenchini@icloud.com (M.M.); 2 Cellular Signalling Laboratory, Department of Biomedical and Neuromotor Science, University of Bologna, 40126 Bologna, Italy; eleonora.ceneri2@unibo.it (E.C.); matilde.follo@unibo.it (M.Y.F.); 3 Department of Medical and Surgical Sciences for Mothers, Children and Adults, University of Modena and Reggio Emilia, 41124 Modena, Italy; emma.bertucci@unimore.it

**Keywords:** neurons, bone, muscle, NMJ, osteosarcopenia

## Abstract

Osteosarcopenia is a widespread geriatric condition resulting from the coexistence of osteoporosis and sarcopenia, where the connection between bone and muscle is, in part, driven by bone–muscle crosstalk. Given the close, reciprocal influence of muscle on nerve, and vice versa, it is not surprising that there are corresponding aging changes in the biochemistry and morphology of the neuromuscular junction (NMJ). Indeed, degeneration of motor neurons and progressive disruption of the neuromuscular connectivity were observed in old age. Extracellular vesicles (EVs) derived from human amniotic fluid stem cells (hAFSC), exhibiting antioxidant properties, which can also explain their anti-aging and cytoprotective effects, can be considered as potential treatment for age-related diseases. To study cell interactions under both healthy and pathological conditions occurring in musculo–skeletal apparatus, we developed a three-culture system exploiting the use of well-known transwell supports. This system allows both myotubes and neurons, eventually treated with EVs, and osteoblasts, induced to osteoporosis, to interact physically and biochemically. Collectively, this method allowed us to understand how the modifications induced in osteoblasts during bone disorders trigger a cascade of detrimental effects in the muscle and neuron parts. Moreover, we demonstrated the efficacy of hAFSC-EVs in preventing NMJ dysfunction, muscle atrophy, and osteoblast impairment.

## 1. Introduction

It is estimated that by 2050, the population over the age of 60 will double around the world. As the size of the older population increases, so does the occurrence of age-related osteoporosis and sarcopenia, which often develop equally in the same patients [1]. Osteoporosis is a common systemic skeletal disorder characterized by low bone mass and increased bone porosity, along with both structural and micro architectural deterioration of bone tissue. Concomitant with osteoporosis, aging also results in a lower muscle mass (atrophy), known as sarcopenia, that may be caused by multiple factors, such as increased muscle fibrosis, chronic inflammation, and increased levels of reactive oxidative species, among others [2]. While the molecular causes of sarcopenia remain to be fully elucidated, recent findings have implicated the neuromuscular junction (NMJ) as being an important locus in the development and progression of that malady. This synapse, which connects motor neurons to the muscle fibers that they innervate, has been found to degenerate with age, contributing both to senescent-related declines in muscle mass and function [3]. Regrettably, these complex connections among bone, muscle, and nerve remain unclear both in physiological and in pathological conditions. In general, such disorders are difficult to investigate and to understand completely, especially in experimental in vitro models, since this implies a multi-tissue system capable of distinguishing the specific role of each component.

Studying cell interactions in healthy and pathological conditions occurring in musculo–skeletal apparatus requires setting up a new, optimized in vitro model, allowing the isolation of cellular compartments for region-specific analyses. Indeed, the molecular mechanisms that cause both muscle and bone loss, namely osteosarcopenia, are still unclear, and how skeletal muscle cells send retrograde signals to motor neurons (MNs) represents an intriguing field of research.

With the purpose to study the perturbation in NMJs occurring in muscle atrophy, besides bone side defects, an ideal model would contain MNs, myotubes, and osteoblasts to better recapitulate the human disease pathology.

Thus, we developed a three-culture system exploiting the use of well-known transwell supports, in which osteoblasts could be treated separately from muscle and neuron cells. This model is useful since it allows us to perform several analyses. The cell samples were analyzed with histological or immunofluorescence staining since the osteoblast part is seeded onto coverslips, and the muscle–nervous system was processed and visualized with confocal microscopy in order to analyze the neurites passing through the transwell net and contacting myotubes. NMJ formation and morphology were examined focusing on the mitochondrial organization. Indeed, it has been shown that in aging neurons, there were fewer axonal mitochondria, but they were longer and thicker, correlating to alterations in mitochondrial-shaping proteins underlying fission and fusion [4], These age-dependent alterations in mitochondrial structure and function play a crucial role in the increased vulnerability of axon function. Moreover, at the presynaptic terminal, mitochondria are vital to the process of neurotransmitter release by supplying highly demanded ATP and by buffering local calcium content. Importantly, among neuronal mitochondria, those located at the synaptic sites appear to be the most susceptible to regulation by extracellular signals [5].

Aside from physical interaction, additional modifications in the protein expression have been explored by Western blot and RT-PCR approaches. In particular, we tested the mRNA and protein expression levels of osteoporosis, muscle differentiation, atrophy, and neurotransmitter exocytosis markers [6,7,8].

Furthermore, the conditioned medium was collected to analyze the modifications in the secretome produced by the cells. Muscles release various secretory factors, including myokines, peptides, growth factors, and hormones, which can influence bone health independently of mechanical loading. Several of them, such as myostatin, FGF-2, IL6, IL7, and IL15, have been implicated in bone–muscle crosstalk, either positively or negatively [9]. Likewise, the skeleton communicates bidirectionally with other organs through the release of proteins that derived from bone cells [10]. These osteokines secreted by osteoblasts, i.e., FGF-23, osteocalcin (OCN), Sclerostin, were detected and monitored.

Given the complexity of osteosarcopenia, and its frequent occurrence in aging, it is difficult to find a unique pharmacological solution. Leftover discarded samples of human amniotic fluid have been identified as a valuable source of stem cells, with promising potential in regenerative medicine and tissue engineering. Ethical concerns associated with their isolation are minimal, since they can be obtained from either a leftover sample of routine prenatal screening amniocentesis, during the II trimester of gestation (fetal hAFSC), or from amniotic fluid discarded as clinical waste in III trimester-scheduled C-section procedures (perinatal hAFSC). In recent years, hAFSCs have been proposed as potential therapeutics for human tissue repair and regeneration given the encouraging evidence obtained from experimental disease models [11]. Moreover, we demonstrated how hAFSCs can induce bone and muscle regeneration [6,12], showing an indirect effect on NMJ perturbation in a muscle atrophic model [12].

Given the important role of oxidative stress and mitochondrial dysfunction in the pathogenesis of many age-related clinical conditions, including osteosarcopenia and NMJ impairment, the treatment with a biological system, containing antioxidant effects, could be useful to counteract this multi-tissue pathology. Based on this consideration, we tested the therapeutic potential of a treatment with EVs in the muscle/neurons compartment affected by the presence of osteoporotic bone cells.

## 2. Results

### 2.1. Setting of the Experimental Model of Triple Culture

**Figure 1:**
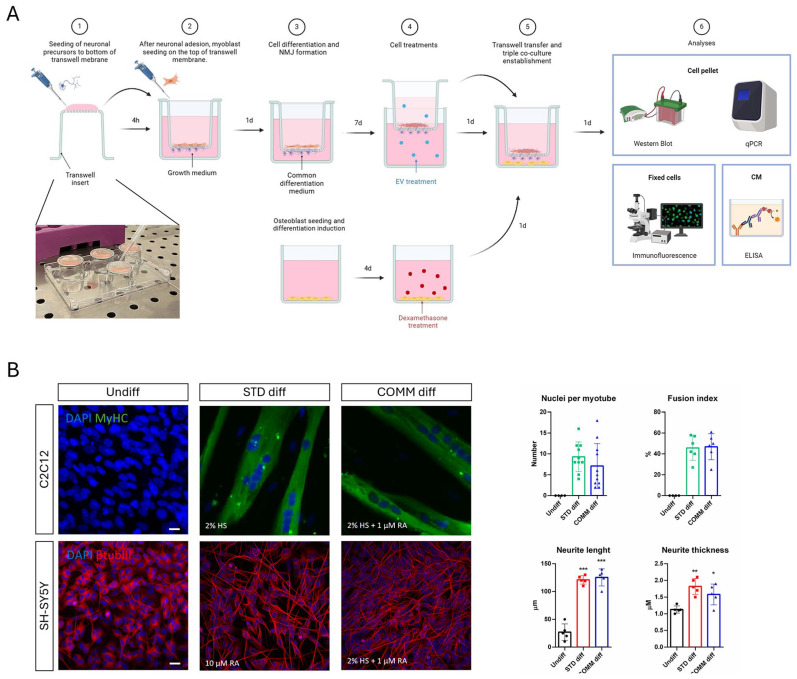
Experimental design and optimization. (**A**) Schematic overview of the co-culture structure and treatment. Created with BioRender.com. (**B**) Immunofluorescence images of differentiation towards myogenic (MyHC) and neurogenic (βtubIII) features obtained with standard (STD) and common (COMM) differentiation media. Nuclei are stained with DAPI (blue). Scale bar: 10 µm. The graphs represent the following: the mean ± SD of number of nuclei/myotube; how many nuclei are involved in myotubes compared to total number of nuclei (fusion index); the length of neurites; the neurite thickness. * *p* value < 0.05; ** *p* value < 0.01; *** *p* value < 0.001.

The scheme in Figure 1A summarizes the experimental protocol used to obtain a triple culture in which myoblast (C2C12) could be seeded and induced to become myotubes, while neuroblastoma cells (SH-SY5Y) were seeded as well and differentiated toward neurons extending their neurites through a net with pores of 1 µm, forming NMJs with myotubes.

In this model, osteoblasts precursors (HOB) were seeded on the bottom of the well and differentiated. Before introducing the transwell insert containing both myotubes and neurons, HOB cells were pharmacologically treated with Dexamethasone (Dexa) to induce an osteoporotic-like condition. The transwell insert was prepared in two phases: it was initially placed upside down to allow seeding of SH-SY5Y cells, and then, after neuronal attachment, the transwell was put upright and C2C12 cells were seeded on the apical side. Both cell lines were cultured in a common differentiation medium.

Therefore, the first aim of this study was to find a common differentiation medium for both neurons and myotubes, since both the cell lines were seeded in the same support (Figure 1A): We demonstrated that a medium, containing retinoic acid (1 μM RA) and horse serum (2% HS), is able to drive an efficient differentiation of both cell types. Figure 1B shows that the number of nuclei per myotube, the fusion index (the ratio of the nuclei number in myocytes with two or more nuclei versus the total number of nuclei), as well as the extension and the thickness of neurites are similar in both the differentiation media.

### 2.2. Analysis of the Effect of EV-Treated Myotubes on Osteoporitic Osteoblasts

**Figure 2:**
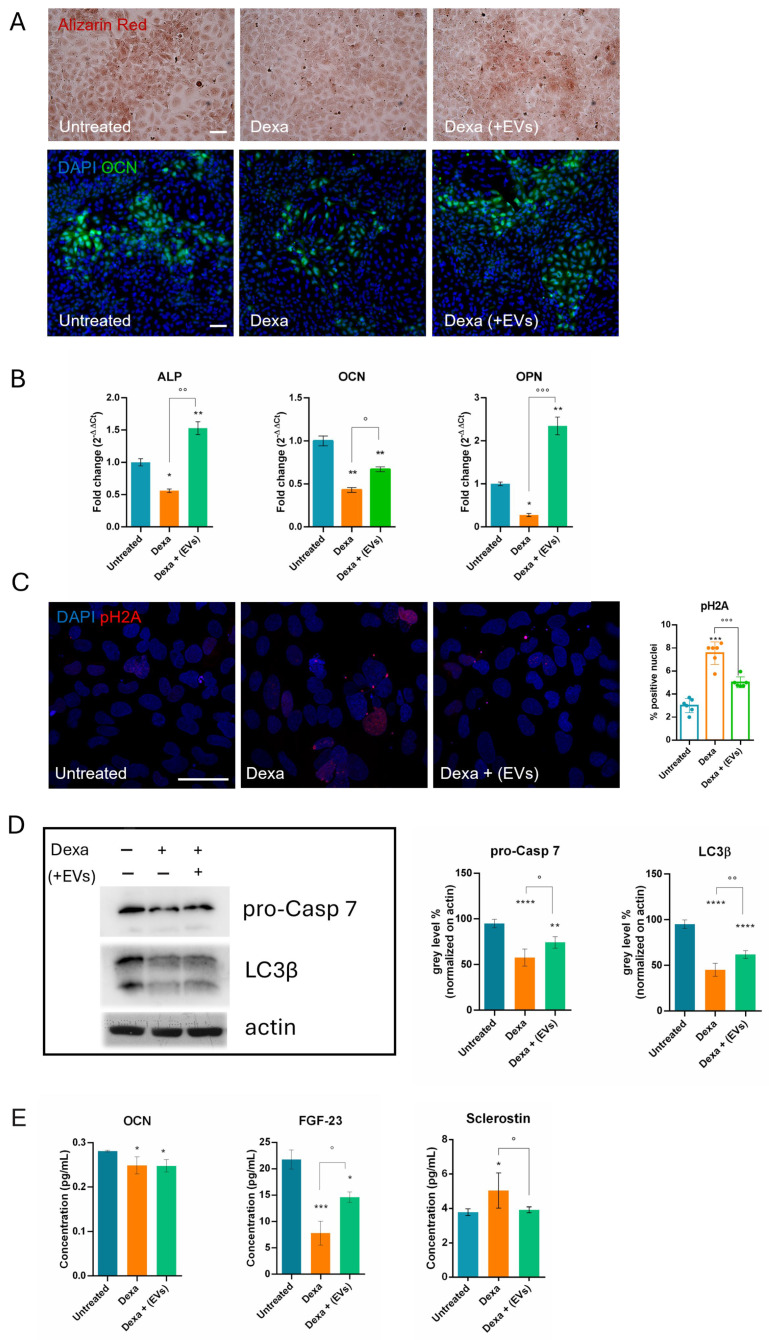
Effect of neuro-myo-EV exposure on cell differentiation and apoptotic/autophagic pathways of HOB treated or untreated with Dexamethasone. (**A**) Representative images of Alizarin Red staining and IF images labeled with DAPI (blue) and OCN (green), signals of HOB cells in triple culture, incubated or not with neuro-myo-EVs (Dexa + (EVs)), and Dexa, are shown. Scale bars: 100 μm. (**B**) Gene expression comparisons of differentiation markers among HOB (Untreated), Dexa 20 µM (Dexa), and Dexa + neuro-myo-EVs (Dexa + (EVs)). °,* *p* value < 0.05, **,°° *p* value < 0.01, °°° *p* value < 0.001. (**C**) Representative IF images labeled with DAPI (blue) and pH2A (red) signals of the same samples of HOB cells. Scale bar: 50 µm. The graph represents the mean ± SD of the percentage of red positive. ***,°°° *p* value < 0.001. (**D**) Western blot analysis of HOB cells treated or not with Dexa and Dexa + (EVs), then revealed with anti-LC3β and anti-pro-caspase 7. The graphs represent the mean ± SD of densitometric analysis of 3 experiments, normalized to actin values. ° *p* value < 0.05; **,°° *p* value < 0.01; **** *p* value < 0.0001. (**E**) ELISA tests for OCN, FGF-23, and Sclerostin on the conditioned medium obtained under the three conditions. *,° *p* value < 0.05; *** *p* value < 0.001.

Then, we demonstrated that osteoblasts, even under triple culture conditions, show osteoporotic characteristics when pre-treated with Dexa, as we showed in a previous study where HOB were in single culture. In particular, we observed a decrease in calcium deposition in extracellular matrix (Alizarin red) and intracellular osteocalcin (OCN) staining (Figure 2A). This last data was confirmed by RT-PCR analysis; other two osteogenic markers, namely osteopontin and phosphatase alkaline (ALP), showed a similar trend in the presence of Dexa (Figure 2B).

Data as result of ELISA assays on the secretome contained in the conditioned medium, obtained from the triple culture system, confirmed a reduction in OCN. Furthermore, the level of the osteokines Sclerostin and FGF-23 (Fibroblast Growth Factor 23) were also significantly modulated after the treatment, confirming the induction of an osteoporotic phenotype.

Western blot and IF analyses (Figure 2C,D) demonstrated that differentiated HOB cells, exposed to Dexa prior to the co-culture with myotubes and neurons, showed a decrease in the pro-caspase 7 protein (pro-Casp 7), indicating an apoptotic process occurring, and an increase in pH2A staining, indicating DNA damage. These events were inhibited in the EVs-treated co-culture.

We also observed in HOB cells, exposed to Dexa, a reduction in LC3β, which was partially restored by the co-culture with myotubes and neurons treated with EVs. This could represent an alteration of autophagic flux, indicated by reduction in LC3-II/LC3b, associated with impaired osteoblastic function in osteoporosis models [13].

Interestingly, pre-treatment with EVs, applied solely to neurons and myotubes, led to an improvement of bone degeneration markers; the levels of the latter two osteokines were restored (Figure 2E), suggesting the existence of a cross-communication mechanism between muscle and bone.

### 2.3. Effect of EV-Treatment on Myotubes Exposed to Osteoporitic Osteoblasts

**Figure 3:**
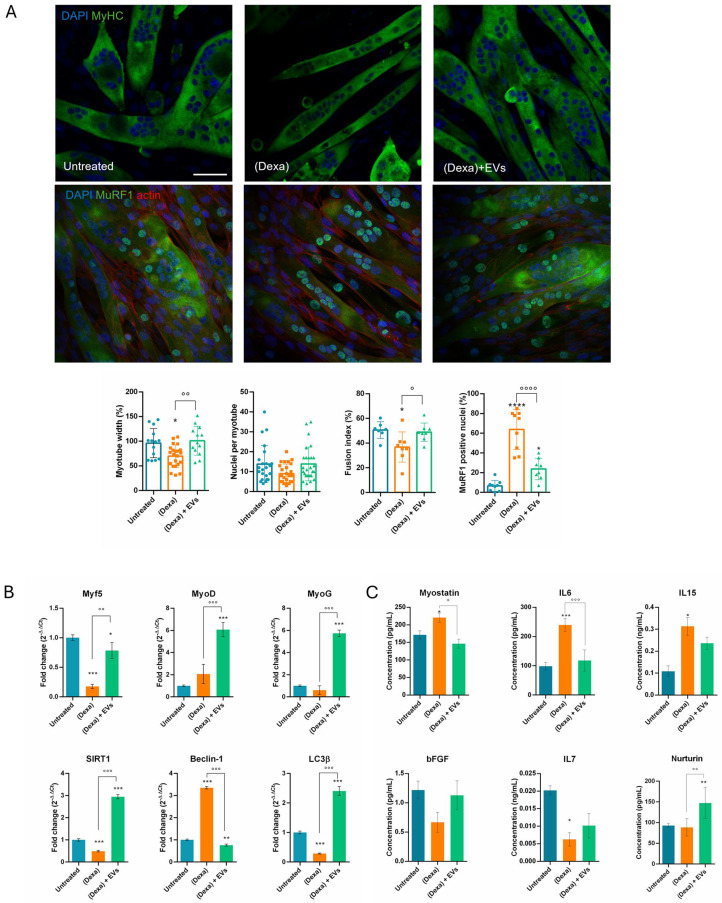
Effect of human AFSC-EV supplementation on myotubes in culture with osteoporotic osteoblasts and neurons. (**A**) Representative images of C2C12-derived myotubes, treated or not with Dexa-HOB (Dexa) and AFSC-EVs ((Dexa) + EVs), stained with DAPI (blue) for nuclei, myosin heavy chain (MyHC) (green), or MuRF1 (green) and actin (red). Scale bars: 100 µm. Graphs relative to analysis of myotube thickness, fusion index%, nuclei per myotube, and percentage of positive nuclei for MuRF1. Graph data are the mean SD (3 biological replicates, 4 fields for each replicate). *, ° *p* value < 0.05; °° *p* value < 0.01; ****, °°°° *p* value < 0.0001. (**B**) Gene expression comparisons of autophagic pathway markers and differentiation markers among C2C12 (Untreated), treated or not with Dexa-HOB and AFSC-EVs. * *p* value < 0.05; **, °° *p* value < 0.01; ***, °°° *p* value < 0.001. (**C**) ELISA tests on the conditioned medium obtained under the three conditions. *,° *p* value < 0.05; **, °° *p* value < 0.01; ***, °°° *p* value < 0.001.

The muscle part was at first analyzed for the molecular and morphological aspects. In the presence of triple culture where osteoblasts had been pre-exposed to Dexa, the myotubes showed an increase in the MuRF1 presence inside the nuclei, a marker of atrophy, and they were thinner, the fusion index was reduced, and the number of nuclei per myotubes as well (Figure 3A). The pre-treatment of myotubes with EVs prevented these modifications.

The expression profile of myotubes in the presence or absence of osteoporotic osteoblasts was investigated. Markers of autophagic process, such as SIRT1, Beclin, and LC3β, were modulated and the EVs reverted all these alterations (Figure 3B). The observed decrease in LC3β alongside an increase in Beclin is unusual but may suggest an incomplete or dysfunctional activation of autophagy [14].

We analyzed the expression of the following myogenic transcription factors (MRFs) that regulate skeletal muscle formation and regeneration: Myf5, which is expressed early in myoblasts and muscle precursors [15]; MyoD, which plays a role in the plasticity of skeletal muscles [16]; and MyoG (MyoGenin), involved in the terminal stages of differentiation into myotubes [17]. The RT-PCR analysis demonstrated that these myogenic transcription factors decrease or at least remain unaltered after the co-culture with osteoporotic osteoblasts, while the pre-exposure to EVs induced a substantial increase in all three MRFs (Figure 3B).

Considering the crosstalk and the paracrine mechanisms between muscle and bone, myokines can be categorized into two types: bone formation factors and bone resorption factors. The forming type includes fibroblast growth factor 2, also known as basic fibroblast growth factor (bFGF). Its secretion was reduced when osteoblasts were induced into an osteoporotic state (Dexa); however, pre-treatment with EVs prevented this decline (Figure 3C).

ELISA tests on the conditioned medium also showed modulation of Myostatin, a member of the TGF-superfamily known to inhibit osteoblast differentiation and activate osteoclast maturation. Its activity can result in compromised bone structure, bone density, and contractile properties. Indeed, its levels increased under the condition of co-culture with osteoporotic osteoblasts, while pre-exposure to EVs completely reduced this effect.

Interleukin (IL) families are pro-inflammatory mediators secreted by various cell types across the body. Several ILs, including IL6, IL7, and IL15, were identified as myokines. In our experimental condition, we observed a production rise in IL6 and IL15 by myotubes when in co-culture with osteoporotic osteoblasts, and a decline of IL7. All these modulations were at least counteracted by EVs.

Finally, we tested the concentration of Neurturin, a muscle-secreted molecule that acts retrogradely on the motor neuron, stimulating axonal branching and NMJs formation [18]. Interestingly, the presence of EVs stimulated the secretion of this factor (Figure 3C).

### 2.4. Osteoporotic Osteoblasts Affect Neuromuscular Interaction: Effect of Stem Cell EVs

**Figure 4:**
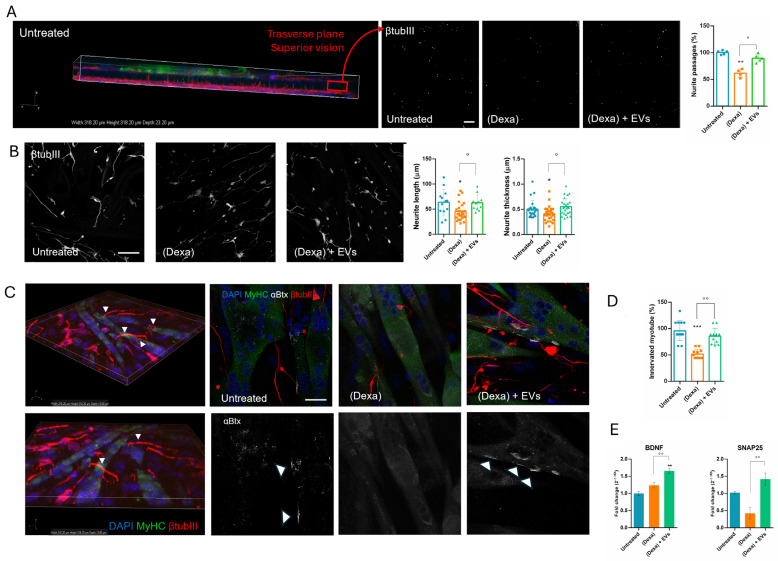
Effect of human AFSC-EV supplementation on the interaction between myotubes and neurons, in culture with osteoporotic osteoblasts. (**A**) Representative 3D image of the transwell net, shown in its thickness, stained with DAPI (blue), βtubIII (red), and MyHC (green) signals. Black and white images show the βtubIII staining (white) through the net under the three conditions. Scale bar: 50 µm. The graph represents the number of neurite passages. ° *p* value < 0.05; ** *p* value < 0.01. (**B**) Representative IF images of the top of the net where neurites of neuron cells, incubated or not with EVs and in culture with osteoporotic osteoblasts, were stained with βtubIII (white). Scale bar: 25 µm. Graphs related to analysis of the neurite length, thickness and passage. *,° *p* value < 0.05. (**C**) On the left, representative 3D images of the transwell net, shown in its top, stained with DAPI (blue), βtubIII (red), and MyHC (green) signals. White arrowheads underline the neurite–myotube interactions. The staining with bungarotoxin (αBtx, in white) was added, alone or superimposed, to the samples under the three conditions. Scale bar: 50 µm. (**D**) The graph represents the number of innervated myotubes. °° *p* value < 0.01; *** *p* value < 0.001. (**E**) Gene expression comparisons of neuronal markers among SH-SY5Y in triple culture treated or not (Untreated) with Dexa-HOB and AFSC-EVs. **, °° *p* value < 0.01.

We analyzed the physical interaction between neurons and myotubes by confocal microscopy. Figure 4A shows the 3D projection of the transwell membrane, with myotube cultured on the upper side (green staining for MyHC) and neurons on the underside (red stating for βtubIII). We demonstrated that the neurites can reach the myotubes passing through the membrane of the insert, recapitulating their physiological interaction. These neurite projections appear as white spots within the pores, which are shown in three images on the right side of Figure 4A. Under the (Dexa) condition, the number of neurites crossing the membrane decreased, unlike with the (Dexa) + EVs condition. Moreover, neurite thickness and length were evaluated: Figure 4B shows that both parameters were reduced under the (Dexa) condition, and that the presence of EVs prevented these declines.

NMJ were visualized by bungarotoxin (αBtx) staining, which labels Acetylcholine (ACh) receptors, coupled with the βtubIII for neurites. Under the (Dexa) condition, co-localization of αBtx clusters on myotubes and neurite terminals was reduced, indicating a lower concentration of ACh receptors. However, this effect was mitigated when muscle cells were pre-treated with EVs (Figure 4C). Moreover, the number of myotubes contacted by neurites dropped by 50% in (Dexa) samples, compared to the untreated controls. This decline was reduced by EV treatment (Figure 4D).

The expression of BDNF (Brain-Derived Neurotrophic Factor) and SNAP25 (Synaptosomal-Associated Protein 25 kDa) were analyzed due to their critical roles in neuronal function. BDNF is a neurotrophic factor essential for the survival, growth, and differentiation of neurons [19], while SNAP25 is involved in synaptic vesicle fusion and neurotransmitter release [20]. The presence of EVs stimulated BDNF secretion and restored the decrease in SNAP25 expression observed in (Dexa) samples (Figure 4E).

### 2.5. EVs Prevent Mitochondrial Modifications in Neurons Exposed to Osteoporotic Condition

**Figure 5:**
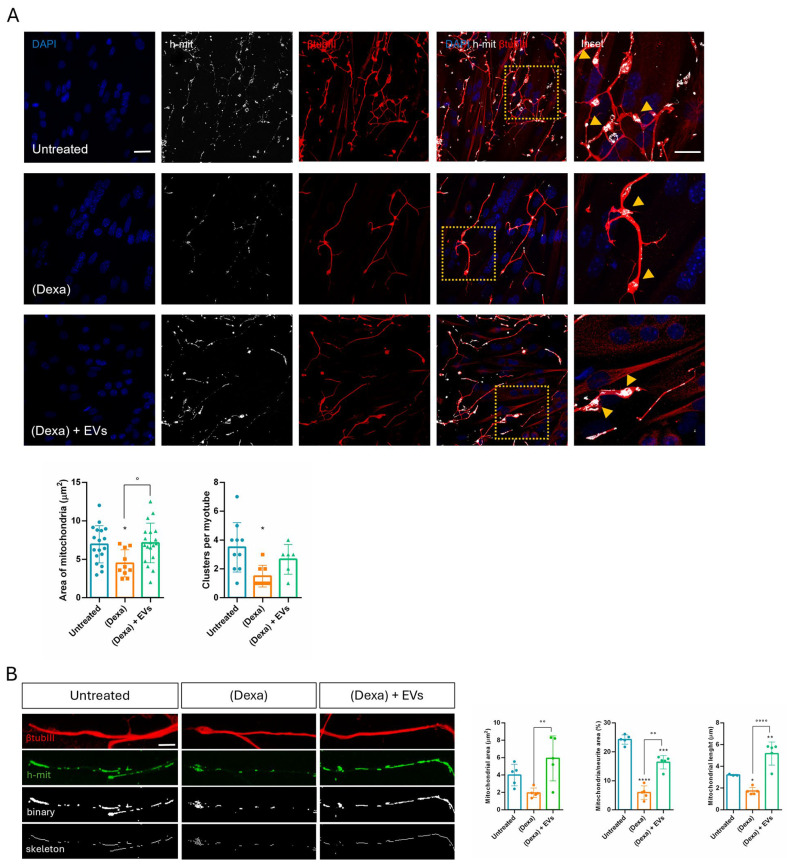
Effect of human AFSC-EV supplementation on mitochondria of SH-SY5Y in co-culture with myotubes and osteoporotic osteoblasts. (**A**) Representative IF images of the top of the net where neurites of neuron cells, incubated or not with EVs and in culture with osteoporotic osteoblasts, were stained with h-mit (white) and βtubIII (red). DAPI staining shows the myotube nuclei. Superimposing and magnification images are shown on the right. Scale bar: 25 µm. Yellow arrowheads underline the mitochondria into the synaptic terminals. Graphs showing the mitochondrial area and the number of clusters. *,° *p* value < 0.05. (**B**) Representative IF images of the top of the net where neurites were stained with h-mit (green) and βtubIII (red), and the mitochondrial distribution through the neurite length was shown in white with binary and skeleton analysis. Scale bar: 5 µm. Graphs showing the mitochondrial area and length, as well as the mitochondrial area per neurite area. * *p* value < 0.05; **, °° *p* value < 0.01; *** *p* value < 0.001; ****, °°°° *p* value < 0.0001.

Morphological analysis of mitochondria in the terminal regions of neurites adjacent to myotubes revealed that mitochondrial area and clustering were abundant in control and (Dexa) + EVs samples, while these parameters are reduced in (Dexa)-only samples (Figure 5A).

Looking at the neurite extension, the mitochondrial area and length were affected under indirect exposure to (Dexa), while EVs’ pre-treatment prevented this phenomenon (Figure 5B). All these observations are consistent with a modulation of fusion/fission process in mitochondria.

## 3. Discussion

In the present study, we designed and optimized a triple culture of neurons, as well as muscle and bone cells, to address the intricate relationship among these tissues under musculo–skeletal pathological conditions. Osteosarcopenia is a geriatric syndrome characterized by the coexistence of osteoporosis (bone loss) and sarcopenia (muscle loss). Muscle and bone are mechanically and biochemically interconnected, and their crosstalk is vital in understanding the pathophysiology of this condition. Additionally, myotubes are also strictly linked to motor neurons, both for physical and functional aspects, through neuromuscular junctions (NMJs). NMJ degradation is both a cause and a consequence of sarcopenia, contributing to the decline in muscle strength and coordination. Aging, inflammation (inflammaging), hormonal decline (e.g., estrogen, testosterone), oxidative stress, and physical inactivity are key drivers of both muscle and bone degeneration. Glucocorticoids, such as Dexamethasone, can exert dose-dependent [21] negative effects on bone tissue by provoking intracellular ROS generation, which are linked to cell death, impaired differentiation, and autophagic pathways, as we previously demonstrated. However, muscle atrophy can be induced in vitro by Dexa as well. Since osteoporosis often precedes or exacerbates sarcopenia through several mechanisms, including the paracrine one, our model required a compartment in which osteoblasts could be induced to osteoporosis separately from the muscle part. Furthermore, to mimic physiological conditions, muscle cells had to be physically separated from neurons yet still contacted by their neurites. To achieve this, myotube and neuron precursors were cultured contemporarily in a shared culture medium able to drive both their differentiation.

For this reason, the first aim of this study was to identify a common differentiation medium to obtain neurons and myotubes. By combining the two main components of neurogenic and myogenic differentiation media—horse serum and retinoic acid—we obtained good neurite extension and classical myotubes as well. In order to generate neuro–muscle interaction with NMJ, we used a transwell system with 1-micron pore membranes, as larger pores would have allowed neurons to invade the muscle compartment, while smaller pores would have prevented neurites from reaching myotubes. We first placed the transwell insert upside down to seed neurons on the underside of the net and then, after neuronal attachment, the transwell was returned to its upright position and myoblasts were seeded on the apical side. This set up offered a practical advantage: during myotube formation, large myotubes may detach so it would be better to have them collected inside the basket.

Meanwhile, osteoblasts were seeded in the multiwell, differentiated, and then exposed to Dexa alone to induce an osteoporotic-like condition. After Dexa removal, the basket net was inserted carrying differentiated neurons and myotubes already interconnected and eventually treated with extracellular vesicles produced by amniotic fluid stem cells. The choice to treat only the neuro–muscle part with EVs and only osteoblasts with Dexa was due to the requirement of investigating the crosstalk between the tissues, so an indirect effect of the toxic or of the therapeutic treatment would be mediated by the cell response. Indeed, these tissues are intricately linked in terms of their anatomy, mechanical functions, and biochemical interactions, and can communicate through paracrine and endocrine-like signaling mechanisms. Bone and muscle are gradually recognized as endocrine organs, which can secrete various cytokines to modulate the tissue homeostasis and remodeling to each other.

The osteoporotic effect induced by Dexa on differentiated osteoblasts (HOB cells) was already set up by our lab; however, it remained unclear whether the presence of the co-culture with neuro–muscle parts after the Dexa treatment could lead to any modifications. Therefore, we also tested under this condition the main markers previously shown to be altered during Dexa exposure. Consistent with earlier observations, we demonstrated the induction of apoptosis, DNA damage, and a block in differentiation and autophagic pathways for osteoblasts. Indeed, a decrease in LC3β suggests a reduced autophagic flux, which compromises the ability of osteoblasts to survive oxidative stress and maintain adequate osteogenic function [13]. More interestingly, although EVs were not directly added to osteoblasts, but only to the neuro-muscle part, a regression of Dexa-induced negative effects on bone cells was observed, demonstrating that the cell crosstalk can act efficiently in conveying the therapeutic message across compartments.

Bone homeostasis is tightly regulated by skeletal stem cell (SSC)-based bone formation and osteoclast-based bone resorption. It has been demonstrated that stem cells with distinct lineage hierarchies facilitate new bone formation [22]. Moreover, investigation of age-related changes in hSSCs found that, throughout adulthood, these cells retain the highest potential for osteochondrogenic development in vitro. However, a significant decrease in hSSC frequency with advanced age was demonstrated, revealing diminished clonogenicity and bone/cartilage-forming potential in older patients. The link between impaired hSSC differentiation and compromised bone regeneration is related to a generation of fibro-stromal tissue, suggesting that dysfunction in hSSCs could underlie pathologically shifted fibrogenic lineage dynamics, thereby impacting the major stem cell source for fracture repair [23]. It would be interesting to investigate if the exposure to EVs derived from young stem cells, such as hAFSCs, could stimulate hSSC to revert the fibrogenic profile during aging.

Although bones do not release secretory factors as muscles do with myokines, they are dynamic tissues that play a crucial role in the endocrine system. In fact, the concept of the skeleton as an endocrine organ has shifted our understanding away from perceiving bone solely as a support and protection structure. Osteokines such as osteocalcin enhance muscle function and insulin sensitivity, while Sclerostin inhibits bone formation and is associated with muscle wasting. FGF-23, in addition to its known role in bone mineralization, is also involved in the regulation of muscle mitochondrial function, contributing to the reduction in reactive oxygen species (ROS) levels, thus strengthening the hypothesis of the existence of a positive feedback loop between the two tissues. Given these premises, it is not surprising that Dexa exposure induces a decrease in OCN and FGF-23 and an increase in Sclerostin. However, only the latter two were restored in the presence of EVs-treated neuro–muscle parts.

On the other hand, it can carry a negative message too, as we observed in the muscle part: indeed, myotube morphology was affected when put together with osteoporotic osteoblasts, showing atrophic features, as well as impairment in the differentiation and autophagic pathways. In particular, myotubes were thinner and MuRF1, marker of atrophy, translocated into the nuclei, as previously reported [24,25]. Furthermore, SIRT1 plays an important part in autophagy in skeletal muscle and inhibits muscle atrophy through this mechanism. In addition, SIRT1 directly deacetylates essential autophagy proteins including microtubule-associated protein light chain 3 (LC3), a key regulator of autophagy [26]. It appears that the cell attempts to activate autophagy (↑ Beclin-1) but fails in its actual execution (↓ LC3β), probably due to the reduction in SIRT1, which impairs autophagic and metabolic activity [14]. During muscle atrophy, a decrease in Myf5 and MyoG can be observed [27]. Both MyoD and Myogenin (MyoG) can influence MyHC content and the metabolic properties of myofibers. MyoD, in this study, remained stable or increased, as in the early phase of atrophy or as a regenerative attempt [28]. Notably, we observed that the presence of EVs upregulated all these markers.

Muscles release various secretory factors, including myokines, peptides, growth factors, and hormones, which can influence bone health independently of mechanical loading. Myostatin inhibits muscle growth and negatively affects bone formation, while IL6 promotes bone resorption. IL7 and IL15 are also secreted by skeletal muscle and can induce bone resorption either directly by inducing osteoclastogenesis [29], or indirectly by acting on effector cells as macrophages and NK cells [30]. Here, we showed that myostatin secretion increased in the presence of osteoporotic cells, justifying the reduction in both myotubes and osteoblasts. The rise in IL6 and IL15 can lead to bone dysfunction; however, a limitation of our experimental model is the absence of osteoclasts or macrophages, therefore we cannot observe this effect. The role of IL7 during atrophy is debated [31]: IL7 secretion from muscle may decrease during muscle atrophy, especially under conditions associated with chronic inflammation, such as in our model. When pre-treated with EVs, these alterations were limited. Moreover, Neurturin, a muscle-secreted factor that acts on muscle fibers and motor neurons to couple their characteristics in a functional way [32], increased in the presence of EVs, suggesting a positive role on neurons.

In parallel, neurons can secrete other molecules such as SNAP25, which is predominantly expressed in mature neurons, and its expression is considered a reliable indicator of the presence and integrity of functional synapses [20]. BDNF expression is highly regulated and varies in different physiological and pathological conditions, making it a sensitive indicator of neuronal status [19]. Interestingly, both their expressions were enhanced still in the presence of EVs. This observation can be linked to the amelioration of the interconnection between neurites and myotubes occurring in samples treated with both EVs and osteoporotic osteoblasts. Indeed, the co-culture with Dexa-osteoblasts reduced the number of neurites passing through the transwell net, the formation of NMJs, and the clustering of Ach-receptors on myotubes.

Sarcopenia is caused by complex and interdependent pathophysiological mechanisms, including aging, neuromuscular junction impairment, and mitochondrial dysfunction. Mitochondria, which are plentiful in neurons along the neurites and the synaptic terminals, play an important role in neuromuscular junction transmission. Here we observed that, in Dexa samples, mitochondria in the synaptic terminals appeared as shorter and rounder or decreased the number of mitochondrial presences in some terminals. These changes can trigger retrograde signaling, leading to motor neuron retraction. Synaptic mitochondrial dysfunction during atrophy compromises neurotransmission, contributes to NMJ destabilization, and may precede or exacerbate motor neuron withdrawal [33]. Similarly, mitochondrial fragmentation occurred along the neurites, likely because atrophy impairs axonal transport of mitochondria to synaptic terminals [34]. Therefore, an imbalance in mitochondrial fusion and fission contributes to NMJ impairment observed in the neuro–muscle part exposed to an osteoporotic environment, but EVs derived from stem cells can prevent this dysfunction.

The cargo of miRNAs and proteins of EVs derived from amniotic fluid stem cells, obtained during full-term C-sections, was in part previously published in our study in 2024 [35], where we already discussed the antioxidant and anticancer roles of the most abundant miRNAs and proteins contained in caesarian EVs. Here, we focused our attention on their role in preserving neuronal mitochondrial function and in ameliorating atrophic muscle cells. Supplementary Figure S1 recapitulates the list of the most abundant miRNAs, while below we highlight the literature supporting the potential efficacy of these EVs in the context of osteosarcopenia pathology because of some of these miRNAs. Moreover, we performed a STRING analysis to examine the EV protein cargo involved in neuronal and synaptic health.

The main synaptic proteins in our list include CYFIP1, CFL1, DLG1/4, NSF, DNM1/1L/2, RIMS1, and STXBP1. Many of them participate directly in critical processes such as cytoskeletal organization within dendritic spines, synaptic vesicle trafficking and fusion, and endocytosis. RIMS1, in turn, is essential for the efficient neurotransmitter release [36,37]. Furthermore, NCAM1 (Neural Cell Adhesion Molecule 1) modulates neuronal survival and interacts with BDNF and FGFR regulating neuronal migration, synaptogenesis, and regeneration [38].

Thanks to this content, we observed a beneficial effect of EVs on neuron-expressed molecules (SNAP25 and BDNF), as shown in Figure 4E.

Looking at the miRNAs list, we noticed that miR-125b, -181a and -34a are the most involved in this scenario, in particular in the mitochondrial balance.

It is reported that miR-125b reduces mitochondrial respiration and promotes elongation of mitochondrial network through BIK and MTP18 silencing, respectively [39]. Moreover, the expression of miR-125b-5p was found down-regulated in both atrophic C2C12 myotubes and denervated muscles. Overexpression of miR-125b-5p protected skeletal muscle samples from atrophy both in vitro and in vivo by targeting TRAF6 through the inactivation of several ubiquitin–proteasome system (UPS)- and autophagy–lysosome system (ALS)-related proteins [40].

Notably, a small pool of nuclear-coded miRNAs was found within mitochondria (mitomiRs), such as mitomiR-181a and -34a. Although their role is still largely unknown, some data suggest that they may modulate the expression of functional mitochondrial proteins and that they can translationally regulate mitochondria-encoded proteins [41]. Bcl-2 has been validated as a target of miR-181a and -34a in different cellular systems. Its physiological roles not only include the inhibition of apoptosis, but also of autophagy and ROS production, as well as the promotion of mitochondrial fusion in non-apoptotic cells [42]. Indeed, overexpression of miR-181a slowed down mitochondrial fission and cell death [43]. miR-181 family gained more attention due to their regulation of processes associated with mitochondrial dynamics and direct targeting of PARK2 and p62/SQSTM1. The overexpression of the miR-181a/b1 cluster was also demonstrated to enhance osteogenesis, through protein synthesis and mitochondrial metabolism, and increased myofiber size and muscle force in skeletal muscle from old mice [44].

We can conclude that we have obtained an easy and reliable system to study, with different approaches, the cell types involved in osteosarcopenia. The alterations in the bone portion drove an atrophic feature in the muscle one. The latter responded to the bone with a paracrine signal as well. It would be a step forward to add the osteoclast cell type to this co-culture in order to follow the bone remodeling aspect too. In vitro studies, such as the present one, are limited by the lack of complexity typical of the human body, therefore in vivo studies, by using animal models, are the next step to validate this therapeutic approach in order to translate into clinical practice. By using in vivo models of osteosarcopenia, it would be easier to evaluate the role of osteoclasts, the cell type missing in our study, as already stated. Moreover, the animal model should be used to assess the optimal EV dose and the method of administration. In vivo experiments could give more information on the role on the whole body on the skeletal tissue aging process; indeed, it would test the levels of a brain-derived osteoanabolic hormone MBH, CCN3, involved in the brain–body crosstalk [45].

Overall, this system is useful to analyze the modification on NMJs, as they are key sites of the cell crosstalk in a bidirectional way in sarcopenia. Indeed, mitochondrial alterations in the synaptic boutons occurred, thus propagating the osteoporotic signal up to the neuron part. EVs, derived from stem cells, can be identified as therapeutic tools since they can interfere with this crosstalk and can prevent the pathological cascade, thanks to their miRNA and protein enrichment.

## 4. Materials and Methods

### 4.1. Amniotic Fluid Stem Cell Isolation and Culture

hAFSCs were obtained from 9 amniotic fluid samples from healthy human donors, their average age being (35.5 ± 2.9), collected during full-term C-sections. In general, the time between collection and processing was kept as short as possible to minimize cell death. First, cells were collected by gradient Ficoll separation, then washed with PBS and centrifuged at 300× *g* for 5 min [35].

The supernatant was discarded, and the pellet was washed again with PBS and dissolved in Ammonium chloride to reach 0.8% to lyse the remaining erythrocytes. Thereafter, the cell solution was incubated at 4 °C for 20 min and centrifuged again. This procedure was repeated until the pellet had a clear color. Afterward, the cells were cultured in culture medium (αMEM), supplemented with 20% (*v*/*v*) fetal bovine serum (FBS), 2 mM L-glutamine, 100 U/mL penicillin, and 100 μg/mL streptomycin (all from EuroClone Spa, Milano, Italy). Once attached, the colonies were visible after 7–10 days and the medium was changed. hAFSCs from full-term Caesarian section were already characterized in a previous study: around 80% of cells were positive mesenchymal stromal markers [35].

### 4.2. Extracellular Vesicles Isolation

hAFSCs were grown in 150 cm^2^ flasks until sub-confluence (around 1.5 × 10^6^ cells). Then, cells were maintained in FBS-free culture medium (18 mL) for 4 days to avoid contamination by EVs from the FBS solution. The collected conditioned medium (CM) was centrifuged at 300× *g* for 10 min at 4 °C to eliminate cellular debris and then concentrated up to 2 mL by using centrifugal filter units with a 3K cut-off (Merck Millipore, Burlington, MA, USA). The concentrated CM was again centrifuged at 10,000× *g* for 30 min at 4 °C, and then the supernatant was ultracentrifuged in polypropylene ultracentrifuge tubes (13.5 mL, Beckman Coulter, Indianapolis, IN, USA) at 100,000× *g* for 90 min at 4 °C in a Beckman Coulter Optima L-90 K centrifuge (SW-41 rotor); the supernatants were discarded and the pellets were resuspended in 13 mL DPBS (Corning, Manassas, VA, USA) and ultracentrifuged again (100,000× *g*, 90 min at 4 °C) [46]. The pellet was resuspended in a ratio of 18 mL to 25 µL of DPBS for subsequent analyses and treatments. After dilution in 1:1000, the size distribution and concentration of EVs were analyzed by nanoparticle tracking analysis using a ZetaView particle tracker from ParticleMetrix (Ammersee, Germany). EV average size was 167 ± 13 nm and the average yield of EVs was 2 × 10^9^/10^6^ cells.

### 4.3. Cell Line Culture

Human pre-osteoblast cells (HOB) were grown in culture medium (αMEM) supplemented with 10% (*v*/*v*) FBS, 2 mM L-glutamine, 100 U/mL penicillin, and 100 µg/mL streptomycin (all from EuroClone Spa, Milano, Italy). Osteogenic differentiation was obtained after 6 days of culture in a differentiation medium composed of αMEM supplemented with 0.5% FBS, 2 mM L-glutamine, 100 U/mL penicillin, and 100 µg/mL streptomycin (all from EuroClone Spa, Milano, Italy), 100 µM 2P-ascorbic acid, and 5 mM β-glycerophosphate (all from Sigma-Aldrich, St. Louis, MO, USA).

The SH-SY5Y human neuron cell line was grown in high-glucose DMEM supplemented with 10% (*v*/*v*) of FBS, 2 mM L-glutamine, 100 U/mL of penicillin, and 100 µg/mL of streptomycin (all from EuroClone Spa, Milano, Italy). Neuronal differentiation was induced in high-glucose DMEM supplemented with 0.5% of FBS and 10 μM all-trans retinoic acid for 7 days.

Murine myoblast cells C2C12 were grown in high-glucose DMEM supplemented with 10% (*v*/*v*) of FBS, 2 mM L-glutamine, 100 U/mL of penicillin, and 100 µg/mL of streptomycin (all from EuroClone Spa, Milano, Italy). Myotube formation was induced, exposing confluent cells for 7 days to a differentiation medium composed of high-glucose DMEM supplemented with 2% horse serum (Sigma-Aldrich, St. Louis, MO, USA).

For muscular and neuronal differentiation into the transwell system, we developed a common differentiation medium composed of high-glucose DMEM supplemented with 2 mM L-glutamine, 100 U/mL of penicillin, and 100 µg/mL of streptomycin (all from EuroClone Spa, Milano, Italy), 2% horse serum, and 1 μM all-trans retinoic acid (all from Sigma-Aldrich, St. Louis, MO, USA) for 9 days. All cells are maintained in a humidified atmosphere with 5% CO_2_ at 37 °C.

### 4.4. Triple Culture Experiments

We developed a three-culture system exploiting the use of well-known transwell supports (Cell culture insert 1.0 µm pore size, 6-well, Falcon, Corning, NY, USA) in which osteoblasts could be treated separately from muscle and neuron cells. On day 1, the insert (put upside down) can be first seeded with neuroblastoma cell line (10^5^ cells/insert) and after cell adhesion (around 4 h); the myoblasts will be put on the other sheet (6 × 10^4^ cells/insert). After 24 h, the common differentiation medium will be added. On day 3, precursors of osteoblasts were seeded on the lower side of 6-well support (5 × 10^4^ cells/insert) and the day after pre-osteoblasts were differentiated. Differentiation media were changed every 48 h. On day 10, osteoporosis could be induced with a pharmacological treatment with 20 µM Dexamethasone (Sigma-Aldrich, St. Louis, MO, USA). At the same time neurons and myotubes were treated with hAFSC-EVs (4 µL/mL). After 24 h, the insert, carrying both myotubes and neurons, could be put in co-culture for 24 h into the well with HOB on the bottom with high-glucose DMEM, 0.5% (*v*/*v*) FBS.

### 4.5. Immunofluorescence and Confocal Microscopy

For immunofluorescence analysis, cells—seeded on coverslips or on the transwell membrane—were processed and confocal imaging was performed using a Leica TCS SP8 confocal laser scanning microscope (Leica, Nussloch, Germany).

Primary antibodies to detect MyHC (myosin heavy chain (MyHC) (In-house, SCIL), β-TubulinIII, pH2A, and h-mitochondria (Merck Millipore, Burlington, MA, USA), osteocalcin and MuRF1 (Santa Cruz Biotechnology, Santa Cruz, CA, USA), p53BP1 (Flarebio Biotech, College Park, MD, USA), and p21 (Novus, Centennial, CO, USA) were used following datasheet recommended dilutions. Bungarotoxin-tetramethylrhodamine (Sigma-Aldrich, St. Louis, MO, USA) was incubated with secondary antibodies according to the manufacturers’ protocol. Alexa secondary antibodies (Thermo Fisher Scientific, Waltham, MA, USA) were used at a 1:200 dilution.

The confocal serial sections were processed with ImageJ-1.51 software to obtain three dimensional projections. The image rendering was performed by Adobe Photoshop CS6 software.

For myotube fusion index, nuclei per myotube, myotube thickness analyses, and NMJ quantifications, MyHC-positive cells containing multiple nuclei were selected as myotubes. Fusion index percentage was calculated as a ratio percentage between the number of nuclei inside myotubes and total nuclei. Myotube thickness was measured using ImageJ software. For NMJ quantification, 40× magnification images of MyHC-positive myotubes were collected and the number of co-localizations between β-TubulinIII (βtubIII) and α-bungarotoxin (αBtx) (Sigma, St. Louis, MO, USA), for Acetylcholine Receptor (AChR) identification, was counted manually through each z-stack, and the number of co-localizations was normalized to the number of myotubes present in the z-stack. Neurite length and thickness analysis was carried out on binary processed images of β-TubulinIII labeling using ImageJ software.

### 4.6. Mitochondria Analysis

Mitochondrial analyses were performed using ImageJ plug-in Mitochondria Network Analysis tool (MiNA) as previously described by Valente et al. [47]. Briefly, confocal images labeled with h-mitochondria (h-mit) (Merck Millipore, Burlington, MA, USA) were pre-processed using unsharp mask, CLAHE, and median filter to enhance image quality. Then, skeletonized images, obtained with the “Skeletonize” ImageJ feature, were analyzed by MiNA plug-in.

Mitochondrial cluster area was carried out on binary processed images of h-mitochondria labeling using the Particle analyzer tool by ImageJ software. Mitochondrial cluster number per myotube was calculated by counting manually the number of clusters on each myotube.

### 4.7. Histological Staining

Fixed monolayer cells were washed with distilled water and then incubated with a 2% Alizarin Red S solution at pH 4.2 for 10 min at RT. Images of histological samples were obtained with a Zeiss Axiophot microscope (Zeiss AG, Jena, Germany), equipped with a Nikon DS-5Mc CCD color camera (Nikon, Tokyo, Japan). 

### 4.8. RNA Isolation and Quantification

RNA was isolated using TRIzolR Reagent (Invitrogen, Waltham, MA, USA) following the manufacturer’s protocol. Starting from 1 μg of the extracted RNA, the cDNA was obtained using SensiFASTTM cDNA Synthesis Kit (Meridian Life Science Inc., Cincinnati, OH, USA) following the manufacturer’s protocol. Real-time PCR was performed using SensiFAST SYBR Hi-ROX Kit following the manufacturer’s protocol (Meridian Life Science Inc., Cincinnati, OH, USA). Real-time PCR reaction was carried out in a total volume of 20 μL loading 250 ng of cDNA and 500 nM of each primer. cDNA amplification was performed by activating the polymerase for 30 s at 95 °C, followed by 40 cycles of 5 s at 95 °C and 30 s at 60 °C. Normalized expression levels were calculated relative to control cells according to the ΔCT method. Primer sequences used in this study are listed below Table 1:

### 4.9. ELISA Assays

Human osteocalcin (Biorbyt, Cambridge, UK), murine myostatin (Cloud Clone, Katy, TX, USA), human Sclerostin, murine neurturin, human FGF-23, murine IL6, murine IL15, murine IL7, and murine bFGF (all from Cusabio Technology, Houston, TX, USA) were quantified in the conditioned medium collected from transwell co-culture by using ELISA assays, according to the manufacturer’s instructions. Samples were run in triplicate. A standard curve was constructed using known concentrations of recombinant human and murine standards.

### 4.10. Western Blot Analysis

Cell extracts were obtained as previously described [48]. Briefly, cells were treated with lysis buffer (20 mM Tris-Cl, pH 7.0; 1% Nonidet P-40; 150 mM NaCl; 10% glycerol; 10 mM EDTA; 20 mM NaF; 5 mM sodium pyrophosphate; and 1 mM Na_3_VO_4_) and freshly added Sigma-Aldrich Protease Inhibitor Cocktail and para-Nitrophenylphosphate (pNPP) at 4 °C for 20 min (all from Sigma-Aldrich, St. Louis, MO, USA). Lysates were sonicated, cleared by centrifugation, and immediately boiled in SDS (Sigma-Aldrich, St. Louis, MO, USA) reducing sample buffer.

Total lysates were loaded onto 4–16% SDS-PAGE. Primary antibodies, prepared as previously reported [49], were raised against the following molecules: Actin (Sigma-Aldrich, St. Louis, MO, USA), OCN (Santa Cruz Biotechnology, Santa Cruz, CA, USA), caspase-7 and LC3β (both from Cell Signaling Technology, Lieden, The Netherlands). Secondary antibodies, used at 1:3000 dilution, were all from Thermo Fisher Scientific (Waltham, MA, USA).

### 4.11. Statistical Analysis

Experiments were performed in triplicate (biological replicates). For quantitative comparisons, values were reported as mean ± SD or SEM based on triplicate analysis for each sample. To test the significance of observed differences among the study groups, One-way ANOVA with Bonferroni post hoc test or Student’s t test was applied. A *p* value < 0.05 was considered statistically significant. Statistical analysis and plot layout were obtained by using GraphPad Prism6 Software v6.

## Figures and Tables

**Table 1:** Primer sequences.

Target	F	R
m-Myf5	AACCAGAGACTCCCCAAGGT	AGCTGGACACGGAGCTTTTA
m-MyoD	AGTGAATGAGGCCTTCGAGA	GCATCTGAGTCGCCACTGTA
m-MyoG	CACTCCCTTACGTCCATCGT	CAGGACAGCCCCACTTAAAA
m-SIRT1	AGGGAACCTTTGCCTCATCTAC	GGTGGCAACTCTGATAAATGAAC
m-Beclin1	TGAATGAGGATGACAGTGAGCA	CACCTGGTTCTCCACACTCTTG
m-MAP1-LC3B	CACTGCTCTGTCTTGTGTAGGTTG	TCGTTGTGCCTTTATTAGTGCATC
m-Actin	CTGGCTCCTAGCACCATGAAGAT	GGTGGACAGTGAGGCCAGGAT
m-GAPDH	CATCAAGAAGGTGGTGAAGC	AAGGTGGAAGAGTGGGAGTT
h-ALP	GCAACTTCCAGACCATTGGC	TCCCACTGACTTCCCTGCTT
h-OPN	ACATCCAGTACCCTGATGCTACAG	TGGCCTTGTATGCACCATTC
h-BDNF	CATCCGAGGACAAGGTGGCTTG	GCCGAACTTTCTGGTCCTCATC
h-SNAP25	CGTCGTATGCTGCAACTGGTTG	GGTTCATGCCTTCTTCGACACG
h-Actin	CACCATTGGCAATGAGCGGTTC	AGGTCTTTGCGGATGTCCACGT
h-GAPDH	GTCTCCTCTGACTTCAACAGCG	ACCACCCTGTTGCTGTAGCCAA

## Data Availability

The datasets presented in this article are not readily available because the data are part of an ongoing study. Requests to access the datasets should be directed to the corresponding author.

## Supplementary Materials

The following supporting information can be downloaded at: https://www.mdpi.com/article/10.3390/ijms26167875/s1.
