# Supplementary figures and images for: Mesenchymal Stromal Cell-Derived Extracellular Vesicles as a Therapeutic Treatment for Osteosarcopenia: Crosstalk Among Neurons, Muscle, and Bone
Source: Int J Mol Sci. 2025 Aug 15;26(16):7875. doi: 10.3390/ijms26167875 (PMC12387062; doi:10.3390/ijms26167875)

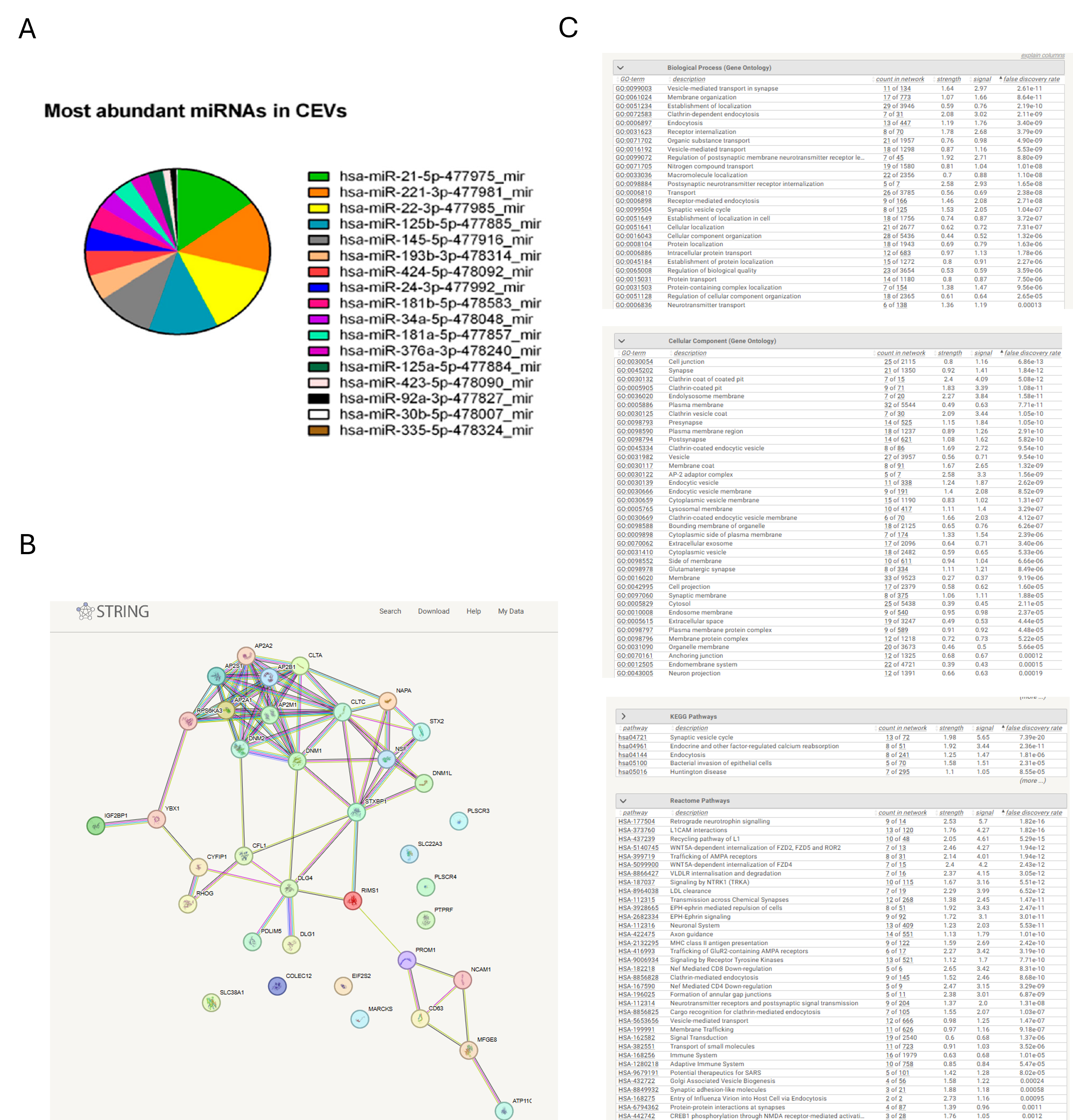

Supplement: Supplementary file 1 [file ijms-26-07875-s001.zip › ijms-3815024-supplementary.tif]
